# BRAF-activated WT1 contributes to cancer growth and regulates autophagy and apoptosis in papillary thyroid carcinoma

**DOI:** 10.1186/s12967-022-03260-7

**Published:** 2022-02-05

**Authors:** Xing Chen, Shan Lin, Ying Lin, Songsong Wu, Minling Zhuo, Ailong Zhang, Junjie Zheng, Zhenhui You

**Affiliations:** 1 Department of General Surgery, Fujian Medical University Provincial Clinical Medical College, Fujian Provincial Hospital, Fuzhou, 350001 Fujian China; 2 Fujian Medical University, Fuzhou, 350001 Fujian China; 3 Department of Paediatric Surgery, Fujian Medical University Provincial Clinical Medical College, Fujian Provincial Hospital, Fujian Medical University, Fuzhou, 350000 Fujian China; 4 Department of Obstetrics and Gynaecology, The Hospital of Changle District, Fuzhou, 350200 China; 5 Department of Ultrasonography, Fujian Medical University Provincial Clinical Medical College, Fujian Provincial Hospital, Fuzhou, 350001 China; 6 Department of Ultrasound, Fujian Medical University Union Hospital, Fuzhou, 350001 Fujian China

**Keywords:** PTC, WT1, Autophagy, Apoptosis, BRAF^V600E^

## Abstract

**Background:**

Papillary thyroid carcinoma (PTC) is one of most prevalent malignant endocrine neoplasms, and it is associated with a high frequency of BRAF gene mutations, which lead to lymphatic metastasis and distant metastasis that promote tumor progression. The molecular mechanism of PTC and the role of BRAF mutation in PTC progression and development need to be further elucidated.

**Methods:**

In this study, a comprehensive bioinformatics analysis was performed to identify the differentially expressed genes and signaling pathways in thyroid cancer patients carrying mutant BRAF. Then, we confirmed the prognostic role of WT1 in thyroid cancer patients. Immunohistochemistry was performed to measure the expression profile of WT1 in PTC tissue. Lentivirus shWT1 was transfected into BRAF^V600E^ (mutant) PTC cells to stably inhibit WT1 expression. CCK-8, EdU, immunofluorescence, colony formation, cell migration, cell wound healing, apoptosis and autophagy assays were performed to assess the biological functions of WT1 in BRAF^V600E^ PTC cells. RNA sequencing, immunohistochemistry and immunoblotting were performed to explore the molecular mechanism of WT1 in BRAF^V600E^ PTC cells.

**Results:**

The results confirmed that “epithelial cell proliferation”, “apoptosis” and “selective autophagy” were closely associated with this BRAF mutant in these thyroid cancer patients. Knocking down BRAF-activated WT1 effectively inhibited the proliferation and migration of BRAF^V600E^ PTC cells. Silencing WT1 significantly inhibited autophagy and promoted the apoptosis of BRAF^V600E^ PTC cells. Mechanistic investigations showed that silencing WT1 expression remarkably suppressed the AKT/mTOR and ERK/P65 signaling pathways in BRAF^V600E^ PTC cells.

**Conclusion:**

All these results indicate that WT1 is a promising prognostic biomarker and facilitates PTC progression and development of cells carrying the BRAF^V600E^ mutation.

**Supplementary Information:**

The online version contains supplementary material available at 10.1186/s12967-022-03260-7.

## Background

Thyroid cancer is one of the most prevalent malignant endocrine neoplasms, with approximately 5% of newly diagnosed cases, with the morbidity and mortality gradually increasing [1]. Papillary thyroid cancer (PTC) accounts for approximately 85% of all thyroid cancers and is the main thyroid malignancy subtype [2]. Despite improvements in current treatment methods, including surgical management, radioiodine therapy, and levothyroxine treatment, some patients experience metastatic and aggressive spread of this cancer, leading to poor clinical outcomes [3]. Therefore, it is necessary to explore the molecular mechanism of PTC progression and search for valuable biomarkers and therapeutic targets to conquer this disease.

In recent years, with the development of high-throughput detection techniques, it has become more convenient to identify promising biomarkers to predict the prognosis and recurrence of various malignancies [4–6]. Based on these techniques, the measurement of gene mutation status, including BRAF, RAS and TERT mutations, also serves as an effective method for exploring regulatory mechanisms and provides a strategy for the individualized treatment of PTC in patients [7–9]. BRAF mutation is the most common genetic lesion in PTC, accounting for 50% of all cases, and the BRAF^V600E^ mutation is the most frequent mutation (95%) among all mutation types [10]. The BRAF^V600E^ mutant activates the MARK pathway and promotes cancer progression in PTC [11]. Targeting the BRAF^V600E^ mutant is a useful method for treating PTC. However, the underlying mechanism of BRAF function in PTC progression remains unknown.

Wilms’ tumor 1 (WT1), located on chromosome 11p13, encodes a transcription factor that includes a proline-/glutamine-rich domain at the N-terminus and four zinc finger motif DNA-binding domains in the C-terminus [12]. It has been reported that WT1 functions as both an oncogene and tumor suppressor in multiple malignancies, playing a critical role in cell survival, proliferation and differentiation [13–15]. The expression level of WT1 also is an independent prognostic factor for a variety of cancers, including breast cancer, colorectal cancer and gynecological cancer, because of its superior predictive performance [16–18]. However, the value of WT1 expression as a prognostic indicator in PTC remains unclear.

Autophagy is a catabolic process by which lysosomes degrade dysfunctional cellular components inside the cell. Three kinds of autophagy have been described to date: microautophagy, macroautophagy and chaperone-mediated autophagy [19]. The autophagy process is closely related to different diseases, such as diabetes, colitis, cardiovascular disorders and cancer [20]. Apoptosis is considered the most common programmed cell death in mammalian cells and is involved in physiological and pathological processes, including normal cell turnover, chemical-induced cell death and regulation of the immune system [21]. Growing evidence suggests that regulating autophagy and apoptosis is an effective cancer treatment because these processes play important roles in PTC progression and development. In a previous study, the expression levels of WT1 were positively correlated with active autophagy in human osteosarcoma cells [22]. WT1 in breast cancer cells has also been shown to contribute to an increased cell proliferation rate and a reduced apoptosis rate [23]. However, the role of WT1 in PTC progression and the underlying mechanism of WT1 in PTC need to be further explored.

In this study, 67 differentially expressed genes correlating with both survival outcome and BRAF mutant were eventually obtained through the analyses of the genes and signaling pathways between PTC patients containing BRAF mutant and BRAF wild-type. Considering these differentially expressed genes, we found that WT1 may be a valuable biomarker for PTC in patients. Then, we analyzed the effect of the WT1 expression level on the proliferation, migration and tumor growth of BRAF^V600E^ PTC cells. In addition, we explored the role of WT1 in regulating autophagy and apoptosis in BRAF^V600E^ PTC cells and searched for the potential mechanism of WT1 action in BRAF^V600E^ PTC cells. This study explored the oncogenic role of WT1 in PTC progression and development, and the results suggested that WT1 may be a novel therapeutic target in PTC patients with BRAF mutation.

## Materials and methods

### Identification of differentially expressed genes (DEGs)

Somatic mutation profiles, mRNA-sequence data and clinical information of thyroid carcinoma (THCA) patients were downloaded from the TCGA database (https://portal.gdc.cancer.gov/). The THCA cohort dataset included 58 nontumor samples and 510 THCA samples (containing 270 wild-type BRAF samples and 240 mutant BRAF samples). Then, we used the “edgeR” software package (version 3.6.3, https://www.r-project.org) to identify differentially expressed genes in THCA patients (the criteria were an absolute log2 fold change (FC) > 1 and adjusted P value < 0.05). Random forest (R package “ranger”) was performed to evaluate the relative importance of various genes in thyroid cancer patients.

### Functional enrichment analysis and GSEA analysis

Kyoto Encyclopedia of Genes and Genomes (KEGG) analysis and Gene Ontology (GO) enrichment analysis were used to confirm the regulatory signaling pathways associated with differentially expressed genes (DEGs) in IHH4 cells with or without WT1 inhibition. We used the “clusterProfiler” R package (version 3.2.11) to identify GO and KEGG signaling pathways. Gene signatures and pathways were obtained from the Molecular Signatures database (MSigDB), GSEA was performed with GSEA software (Version 4.1.0), and P < 0.05 was considered statistically significant.

### Cell culture

BRAF^V600E^ PTC cell lines (including IHH4 cells and BCPAP cells) were purchased from the American Type Culture Collection (ATCC) (Manassas, VA, USA). The IHH4 cells were cultured with a mixture of DMEM and RPMI 1640 in a one-to-one proportion. Then, the mixed medium was supplemented with 2 mM l-glutamine, 10% fetal bovine serum (FBS) and 100 U/ml penicillin. The BCPAP cells were cultured in FK12 medium containing 10% fetal bovine serum (FBS) and 100 U/mL penicillin. The IHH4 cells and BCPAP cells were incubated at 37 °C in 5% CO_2_ at a 95% atmosphere at constant temperature.

### Cell proliferation assay

IHH4 cells and BCPAP cells were cultured in 96-well plates (3000 cells per plate in 200 µl of medium). Then, 20 µl of Cell Counting Kit-8 (CCK-8) reagent (Beyotime, Shanghai, China) was added to every plate and incubated with medium for approximately two hours. The OD450 value was measured with a microplate spectrophotometer (Thermo Fisher Scientific, MA, USA). Cell proliferation was also analyzed with 5-ethynyl-20-deoxyuridine (EdU) agent (Beyotime, Shanghai, China), and the EdU assay was performed according to the manufacturer’s protocol. In addition, each experiment was repeated three times.

### Transwell assay

IHH4 cells and BCPAP cells (2 × 10^5^ cells per plate) were cultured in the upper chamber of 24-well culture plates with 8-mm-pore membrane inserts. In the upper chamber, 200 µl of serum-free medium was added, and 700 µl of medium supplemented with FBS was added to the lower chamber. After 24 h, the cells above the membrane in the upper chamber cleaned off the membrane, and cells below the upper chamber membrane were stained with 0.4% trypan blue. The migrating cells were counted with a light microscope, and each experiment was repeated three times.

### Western blot analysis

Lysates of the IHH4 cells and BCPAP cells were added to SDS-PAGE sample loading buffer (Beyotime, Shanghai, China) and boiled at 100 °C for 8–10 min. Western blotting was conducted as previously described[24, 25]. The following antibodies were showed in supported information, and anti-actin as an internal control. Protein bands were quantified using Image J software and expressed as fold change with respect to mean control values in the same run (defined as 100 or 1, respectively).

### Lentivirus production and transduction

The WT1-shRNA sequences were cloned into the pLL3.7 vector. The sequences of WT1-shRNA used are shWT1#1: 5′-CCGGGTGTCTGCTAATGTAAACTTTCTCGAGAAAGTTTACATTAGCAGACACTTTTTG-3′; shWT1#2: 5′-CCGGTATAAGTACTAGATGCATCACCTCGAGGTGATGCATCTAGTACTTATATTTTTG-3′. Recombinant lentivirus was generated from 293T cells using calcium phosphate precipitation. IHH4 cells were transfected with lentivirus using polybrene (8 µg/ml) and stable knockdown cells were obtained following selection with 1 μg/ml puromycin for 1 week.

### Colony formation survival

A total of 400 cells were seeded into 6-well in triplicates for plate colony formation survival assay. When visible colonies were formed, cells were fixed after 2 weeks by methanol, stained with 0.2% crystal violet solution then photographing for colony formation assays.

### Cell cycle analysis by flow cytometry

Seventy percent pre-cooled ethanol was used to fix a total of 1 × 10^6^ cells then washed with PBS and subjected to cell apoptosis analysis using Cell Cycle and Apoptosis Analysis Kit (Yeasen) following the manufacturer’s instructions. Data were analyzed by FlowJo v10 software.

### Transmission electron microscopy (TEM)

Cells were fixed with 2% paraformaldehyde and 2.5% glutaraldehyde in 0.1 M cacodylate buffer. Cells were then placed in an ice-cold solution of 1% osmium tetraoxide (Electron Microscopy Sciences) with 0.8% potassium tetraoxide and 3 mM calcium chloride. Ultrathin sections were prepared and supported on 75 mesh copper grids followed by Sato lead staining.

### Nude mouse xenograft models

Male BALB/c nude mice (between 4 and 6 weeks old) were purchased from HFKbio (Beijing, China) to establish subcutaneous PTC mouse models. IHH4 cells were treated with or without WT1 shRNA and these IHH4 cells (5 × 10^6^ per mouse) were injected subcutaneously into nude mice. After the subcutaneous tumor length and width reached approximately 2.5 cm × 2.5 cm, the tumor volume was measured every two days until the tumor volume was approximately 1000 cm^3^. All of the animal studies were approved by the Institutional Animal Care and Use Committees of Fujian Medical University.

### Immunohistochemistry assay

The tumor tissue from BRAF^V600E^ PTC xenograft model with or without WT1 knockdown was incubated with 4% paraformaldehyde for approximately 24 h and embedded in paraffin. IHC assays were used to measure the protein levels of WT1 and Ki67, and the manufacturer’s protocol was constructed as described in a previous study [26]. A primary antibody was used. The integrated optical density (IOD) was quantified using ImageJ software (Version 1.8.0) and the mean optical density (MOD) of each sample was calculated.

### Immunofluorescence assay

IHH4 cells and BCPAP cells were cultured in 6-well plates with cover slips. Then, the cells were placed in 4% paraformaldehyde for approximately 30 min, and then, 0.03% Triton X-100 was added and incubated for 20 min. The manufacturer’s protocol for the immunofluorescence assay was followed as previously described [44]. The images were analyzed by a fluorescence microscope and ImageJ software (Version 1.8.0).

### Statistical analysis

Statistical analysis of the experimental results was performed using GraphPad Prism software (Version 6.0) and R software (Version 4.0.3). Differences between two groups were analyzed using Student’s t-test, and all results are shown as the means ± SEM. The statistical significance was set at P < 0.05, and each experiment was repeated three times.

## Results

### The landscape of mutations and BRAF mutations in thyroid cancer patients

**Fig. 1:**
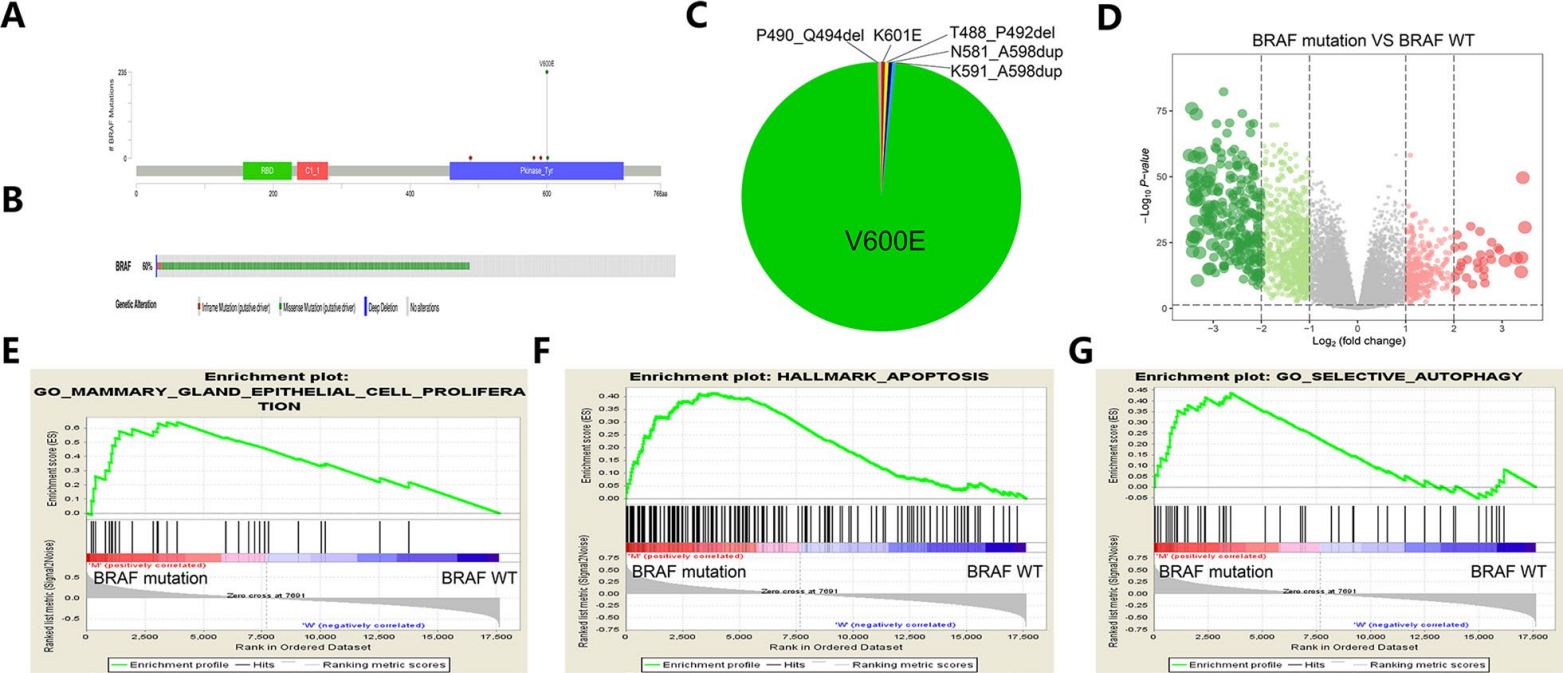
The landscape of mutations and BRAF mutations in thyroid cancer patients. **A**, **B** Somatic mutation of BRAF in thyroid cancer patients. **C** Changes in BRAF protein expression in thyroid cancer patients. **D** Differentially expressed genes in thyroid cancer patients carrying mutant BRAF and wild-type BRAF. **E**–**G** GSEA showing the upregulated signaling pathways associated with BRAF mutation in thyroid cancer patients

In a previous study, gene mutations were found to play important roles in thyroid cancer progression and development [27]. Additional file 1: Fig. S1A, B indicates the landscape of mutations in thyroid cancer patients as identified through the TCGA database. The analysis showed that BRAF is the most frequently mutated gene and is carried by approximately 60% of thyroid cancer patients. In thyroid cancer patients, most variant classifications and SNV classes are missense mutations and C > T mutations. Figure 1A, B shows the landscape of BRAF mutations in thyroid cancer patients according to the cBioPortal database. Figure 1C shows that BRAF^VE400^ is the most common BRAF mutant in thyroid cancer patients. A volcano plot demonstrates the differentially expressed genes (DEGs) in thyroid cancer patients with mutant and wild-type BRAF in the TCGA cohort (Fig. 1D). A gene set enrichment analysis (GSEA) showed the signaling pathways associated with BRAF mutations in thyroid cancer patients. The results verified that “epithelial cell proliferation”, “apoptosis” and “selective autophagy” were closely associated with BRAF mutations in thyroid cancer patients (Fig. 1E–G). These results suggest that the landscape of mutation and BRAF mutation in thyroid cancer patients and signaling pathways, such as epithelial cell proliferation, apoptosis and autophagy pathways, play critical roles in the progression of thyroid cancer with BRAF mutation.

### Identifying the expression and prognostic role of BRAF-activated WT1 in thyroid cancer patients

**Fig. 2:**
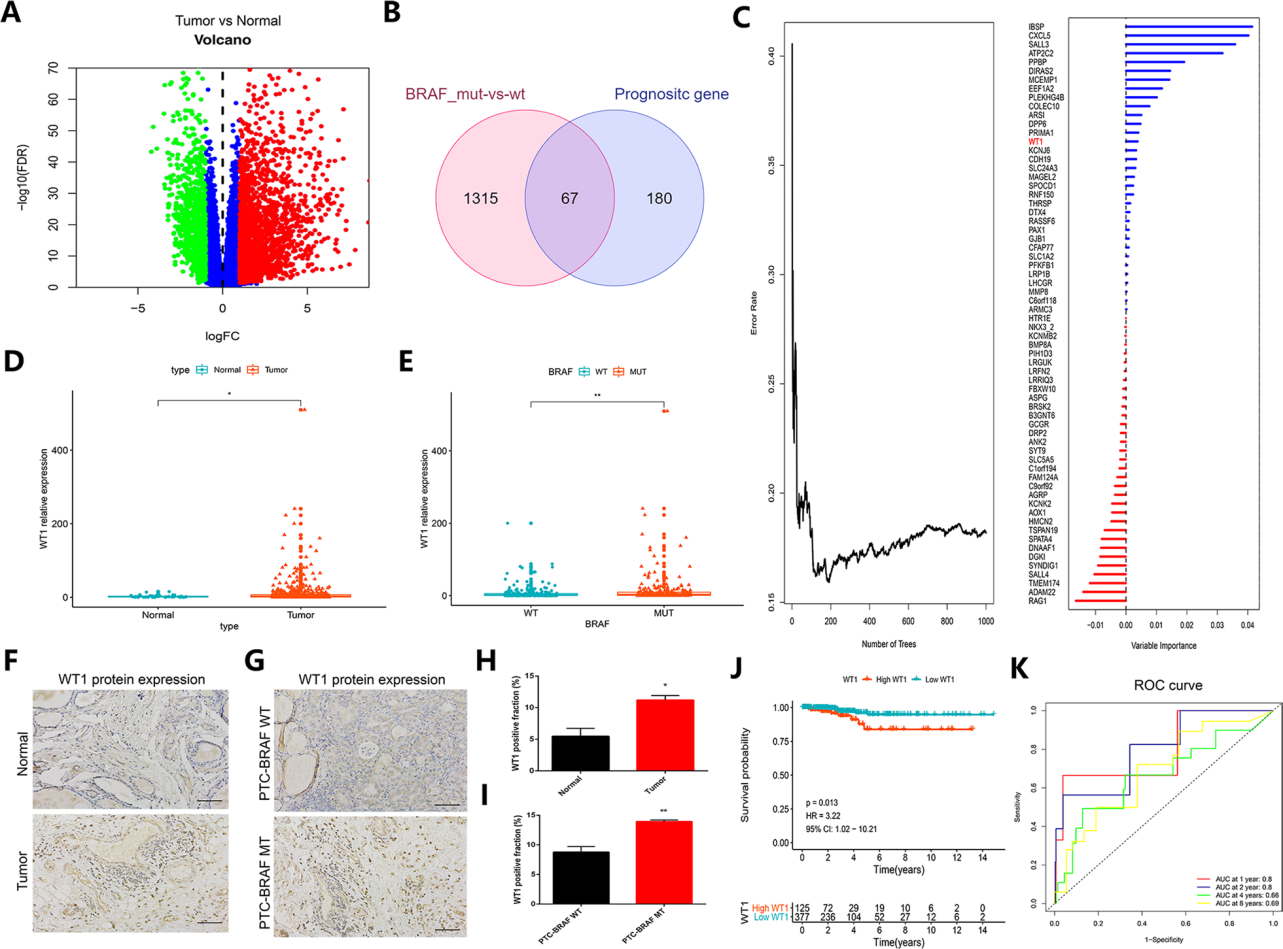
BRAF-activated WT1 is highly expressed in thyroid cancer patients and predicts poor prognosis. **A** The differentially expressed genes between thyroid cancer tissues and normal tissues in the TCGA database. **B** The common regulators among the prognostic genes and DEGs in thyroid cancer patients carrying BRAF-MT and BRAF WT. **C** The importance of 67 common genes in thyroid cancer patients. **D** The expression level of WT1 in non-tumor samples compared with that in THCA samples. **E** The mRNA level of WT1 in THCA samples with or without BRAF mutation. **F** The protein level of WT1 in non-tumor tissues and THCA tumor tissues. **G** The protein expression of WT1 in THCA patients carrying BRAF WT or BRAF MT. **H**, **I** Quantification of immunohistochemistry data of WT1 expression in different groups. **J**, **K** KM plot and ROC curve showing the prognostic value of WT1 in thyroid cancer patients (*P < 0.05, **P < 0.01)

A volcano plot indicates the differentially expressed genes (DEGs) between tumor samples and non-tumor samples of thyroid cancer patients (Fig. 2A). We identified prognostic genes by univariate Cox analysis (P < 0.05). To further explore the components downstream of activated BRAF, the molecules involved with mutant BRAF and common to the prognostic genes and DEGs were identified in thyroid cancer patients (Fig. 2B). Sixty-seven common genes were selected to perform this analysis. The importance of these 67 genes in thyroid cancer patients was analyzed by random forests. The results showed that WT1 is a significantly important regulator in thyroid cancer patients (Fig. 2C). Figure 2D shows that WT1 was remarkably overexpressed in THCA samples compared with non-tumor samples. Interestingly, WT1 was also upregulated in thyroid cancer samples with mutant BRAF compared with thyroid cancer samples with wild-type BRAF (Fig. 2E). Next, immunohistochemistry (IHC) was used to confirm the expression level of WT1 in PTC tissue. The results implied that WT1 was significantly increased in PTC tissue, especially BRAF-mutant PTC tissue (Fig. 2F–I). In addition, higher WT1 expression predicted worse survival time in thyroid cancer patients (Fig. 2J), and the AUC of the ROC curve reached 0.8, 0.8, 0.66 and 0.69 at 1 year, 2 years, 4 years and 8 years, respectively, indicating a superior predictive performance of WT1 for thyroid cancer patients (Fig. 2K). These results suggested that BRAF-activated WT1 may serve as a promising prognostic biomarker in thyroid cancer patients.

### Silencing WT1 expression inhibited the proliferation of BRAF^V600E^ PTC cells

**Fig. 3:**
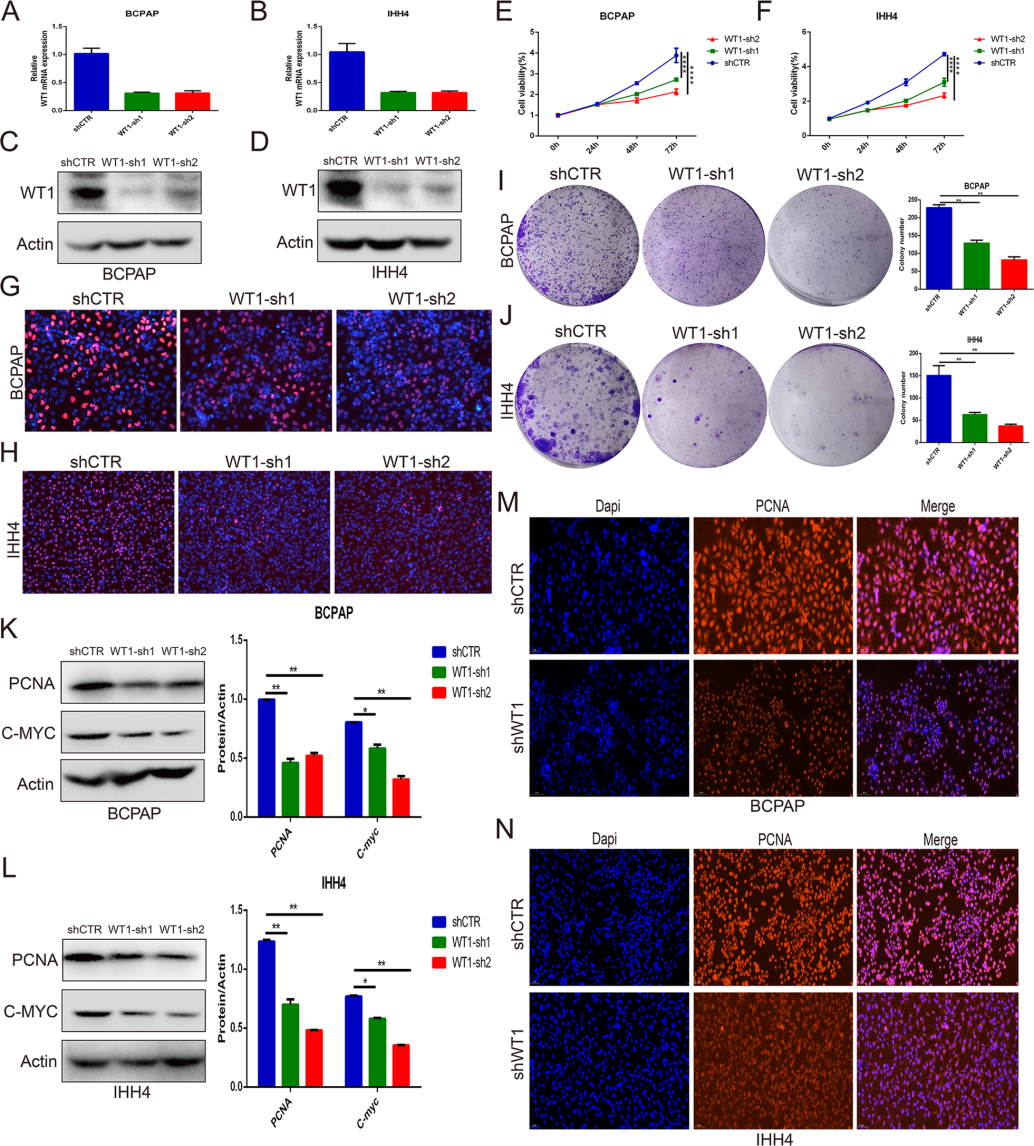
WT1 knockdown inhibited the proliferation of BRAF^V600E^ PTC cells. **A**, **B** Quantitative RT-PCR analysis of the WT1 expression level in the IHH4 cells and BCPAP cells treated with WT1 shRNA. **C**, **D** Western blot assay showing the knockdown efficiency of WT1 in IHH4 cells and BCPAC cells treated with lentiviral WT1 shRNA. **E**, **F** CCK-8 assay indicating the proliferation of IHH4 and BCPAC cells upon WT1 inhibition. **G**, **H** EdU assay indicating the anti-proliferative effect of WT1 shRNA treatment in IHH4 and BCPAC cells. **I**, **J** Colony formation assay showing the proliferation ability of IHH4 and BCPAC cells with WT1 knocked down. **K**, **L** The protein expression levels of PCNA and C-myc in IHH4 and BCPAP cells with WT1 knocked down, as determined by Western blot assay. **M**, **N** Changes in the protein expression levels of PCNA in IHH4 and BCPAP cells after WT1 inhibition by immunofluorescence assay (**P < 0.01, ****P < 0.0001)

To explore the role of WT1 in the proliferation and progression of BRAF^V600E^ PTC cells, BRAF^V600E^ IHH4 cells and BCPAP cells were treated with lentiviral WT1 shRNA. The quantitative RT-PCR and western blot analysis indicated that the expression level of WT1 was remarkably decreased in the IHH4 cells and BCPAP cells treated with WT1 shRNA (Fig. 3A–D). Then, CCK-8, EdU and colony formation assays were performed to measure the proliferation ability of the IHH4 cells and BCPAP cells. The CCK-8 assay indicated that knockdown of WT1 expression significantly suppressed IHH4 and BCPAC cells proliferation (Fig. 3E, F). Similarly, silencing WT1 expression significantly inhibited the proliferation of IHH4 cells and BCPAC cells, as determined by EdU assay (Fig. 3G, H) and colony formation assay (Fig. 3I, J). A western blot analysis showed that WT inhibition notably suppressed the expression levels of PCNA and C-myc in the IHH4 and BCPAP cells (Fig. 3K, L). In addition, immunofluorescence assays implied that the knockdown of WT1 expression effectively decreased the expression of PCNA in the IHH4 cells and BCPAP cells (Fig. 3M, N). These results suggest that WT1 may play an oncogenic role in the proliferation of BRAF^V600E^ PTC cells, namely, IHH4 and BCPAP cells.

### Gene knockdown of WT1 inhibited the migration of BRAF^V600E^ PTC cells

**Fig. 4:**
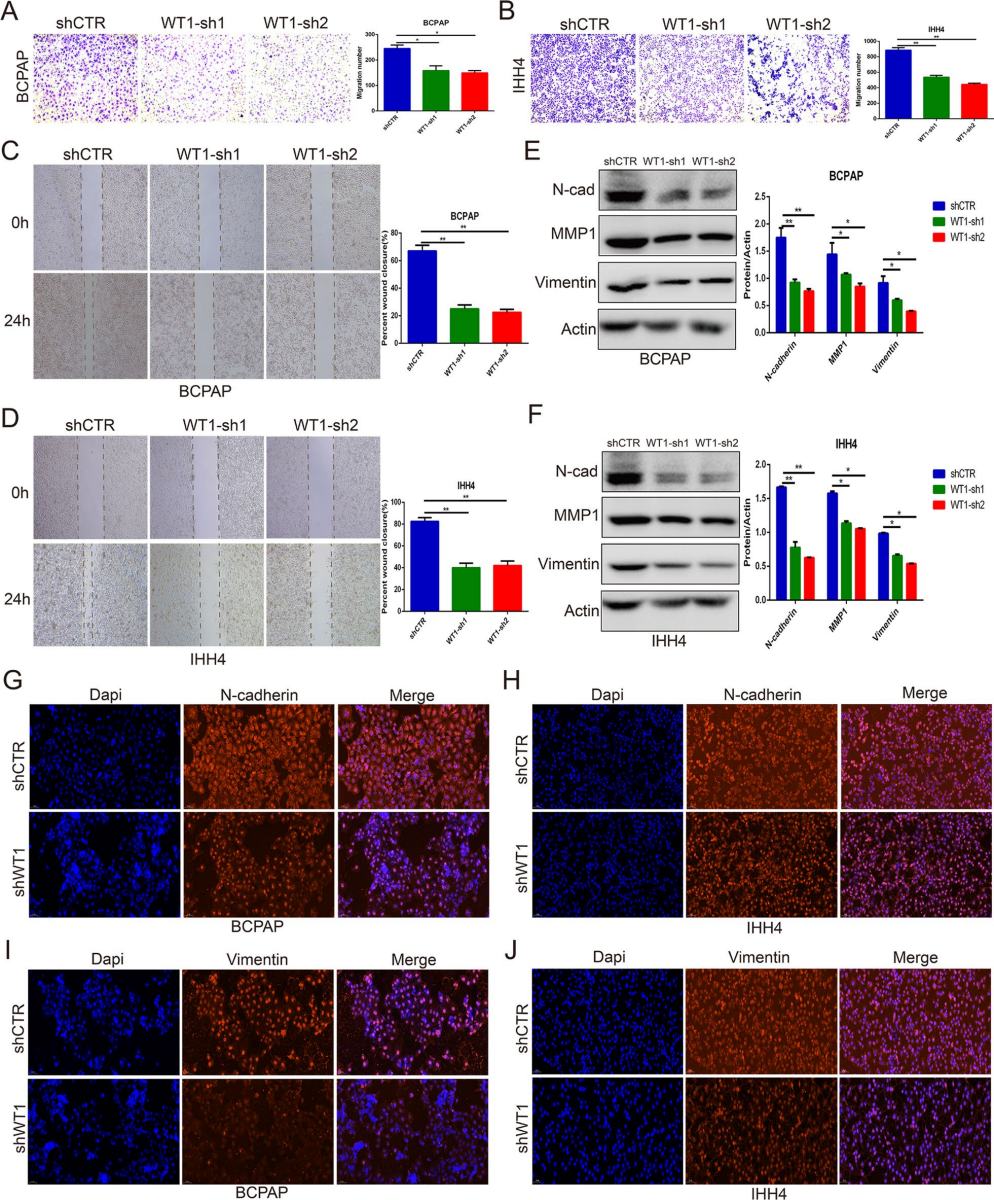
Silencing WT1 expression suppressed the migration of BRAF^V600E^ PTC cells. **A**, **B** Transwell assays showing the number of migrating IHH4 and BCPAP cells after WT1 expression was inhibited. **C**, **D** A healing wound assay indicating suppressed migration of IHH4 cells and BCPAP cells after WT1 expression was inhibited. **E**, **F** Western blot assays confirmed that silencing WT1 expression decreased the protein levels of N-cadherin, vimentin and MMP1 in IHH4 and BCPAP cells. **G**–**J** Immunofluorescence assays showing that WT1 inhibition suppressed N-cadherin and vimentin expression in IHH4 and BCPAP cells (*P < 0.05, **P < 0.01)

To investigate the function of WT1 in regulating BRAF^V600E^ PTC cell migration, Transwell assays and wound healing assays were performed with IHH4 and BCPAP cells. WT1 inhibition remarkably suppressed the migration of IHH4 and BCPAP cells, as indicated by Transwell assay (Fig. 4A, B) and healing wound assay (Fig. 4C, D). Western blot and immunofluorescence assays were also performed to measure the protein levels of proteins involved in the migration process, such as N-cadherin, Vimentin and MMP1. The western blot analysis indicated that knockdown of WT1 expression effectively decreased the protein levels of N-cadherin, Vimentin and MMP1 in IHH4 cells and BCPAP cells (Fig. 4E, F). Similarly, immunofluorescence assays showed that silencing WT1 expression downregulated the protein levels of N-cadherin and Vimentin in IHH4 cells and BCPAP cells (Fig. 4G–J). All the results indicated that WT1 gene knockdown inhibited the migration of BRAF^V600E^ PTC cells.

### WT inhibition suppressed autophagy and promoted apoptosis of BRAF^V600E^ PTC cells

**Fig. 5:**
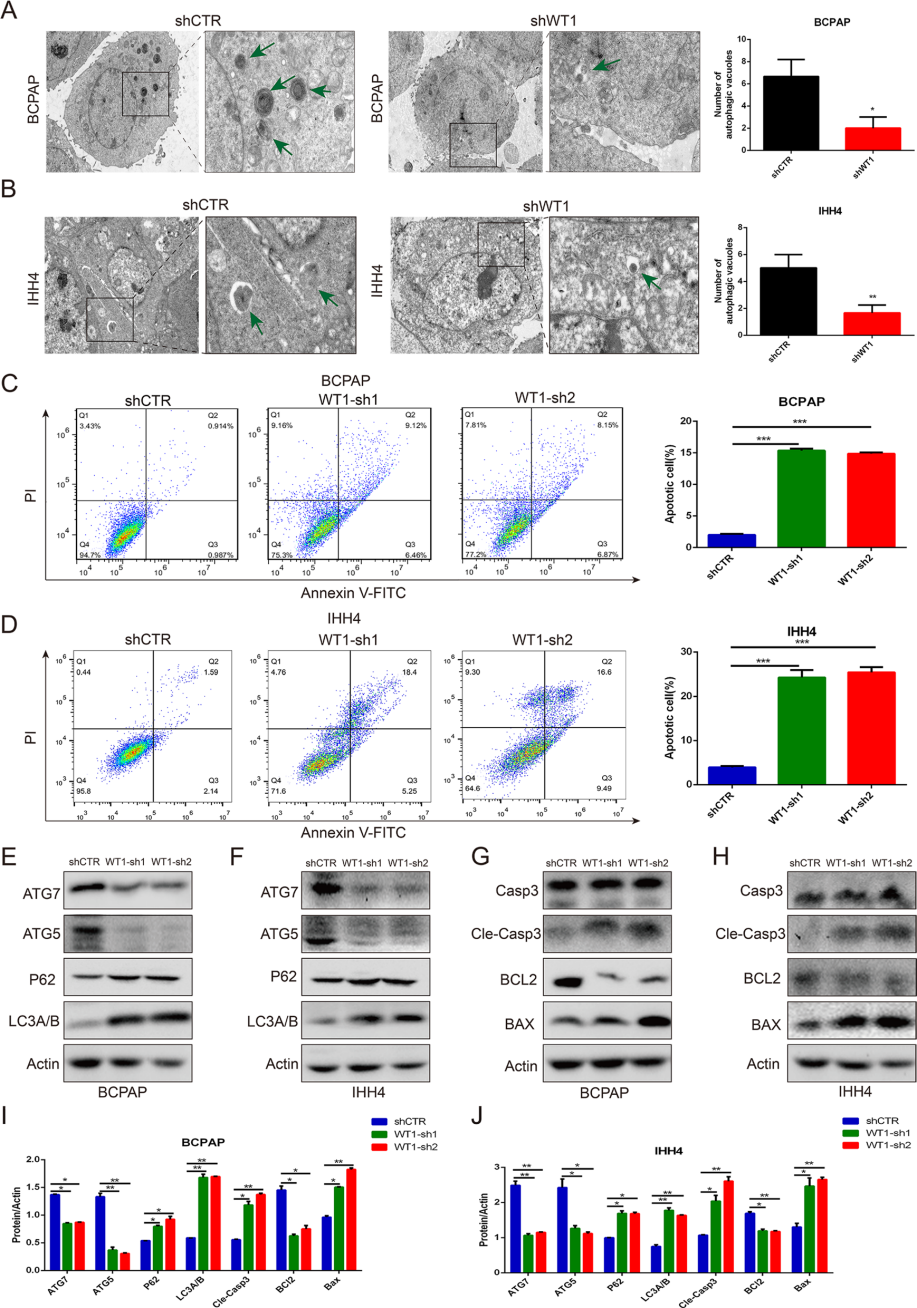
Gene knockdown of WT1 regulated autophagy and apoptosis of BRAF^V600E^ PTC cells. **A**, **B** Transmission electron microscopy was performed to observe the numbers of autophagic vacuoles, including autophagosomes and autolysosomes, in IHH4 and BCPAP cells after WT1 inhibition. **C**, **D** Flow cytometry analysis showing that WT1 inhibition promoted the apoptosis of IHH4 and BCPAP cells. **E**, **F** Western blot analysis showing the protein changes in LC3A/B, P62, ATG5 and ATG7 in IHH4 and BCPAP cells with WT1 knocked down. **G**, **H** Western blot assay showing that WT1 knockdown modulated the protein expression levels of cleaved caspase 3, BCL2 and BAX in IHH4 and BCPAP cells. **I**, **J** Quantification of protein expression of ATG7, ATG5, P62, LC3A/B, BCL2, BAX and cleaved caspase 3 in WT1-silencing IHH4 cells and BCPAP cells (*P < 0.05, ***P < 0.001)

To explore the biological process of WT1 in BRAF^V600E^ PTC progression and development, Transmission electron microscopy (TEM) assays indicated that the number of autophagic vacuoles (autophagosomes and autolysosomes) was significantly reduced in IHH4 and BCPAP cells after WT1 inhibition (Fig. 5A, B). Flow cytometry analysis showed that silencing WT1 expression significantly induced apoptosis of the IHH4 cells and BCPAP cells (Fig. 5C, D).Interestingly, knockdown of WT1 expression notably increased the protein level of P62 and LC3A/B and downregulated the protein levels of ATG5 and ATG7 in the IHH4 cells and BCPAP cells (Fig. 5E, F, I J). A western blot analysis demonstrated that WT1 inhibition effectively enhanced the protein expression of cleaved caspase 3, and BAX and suppressed the protein expression level of BCL2 in the IHH4 cells and BCPAP cells (Fig. 5G–J). These results demonstrate that the altered expression of WT1 leads to changes in autophagy and apoptosis pathways in BRAF^V600E^ PTC cells.

### WT1 activated the AKT/mTOR signaling pathway and ERK/P65 signaling pathway in BRAF^V600E^ PTC cells

**Fig. 6:**
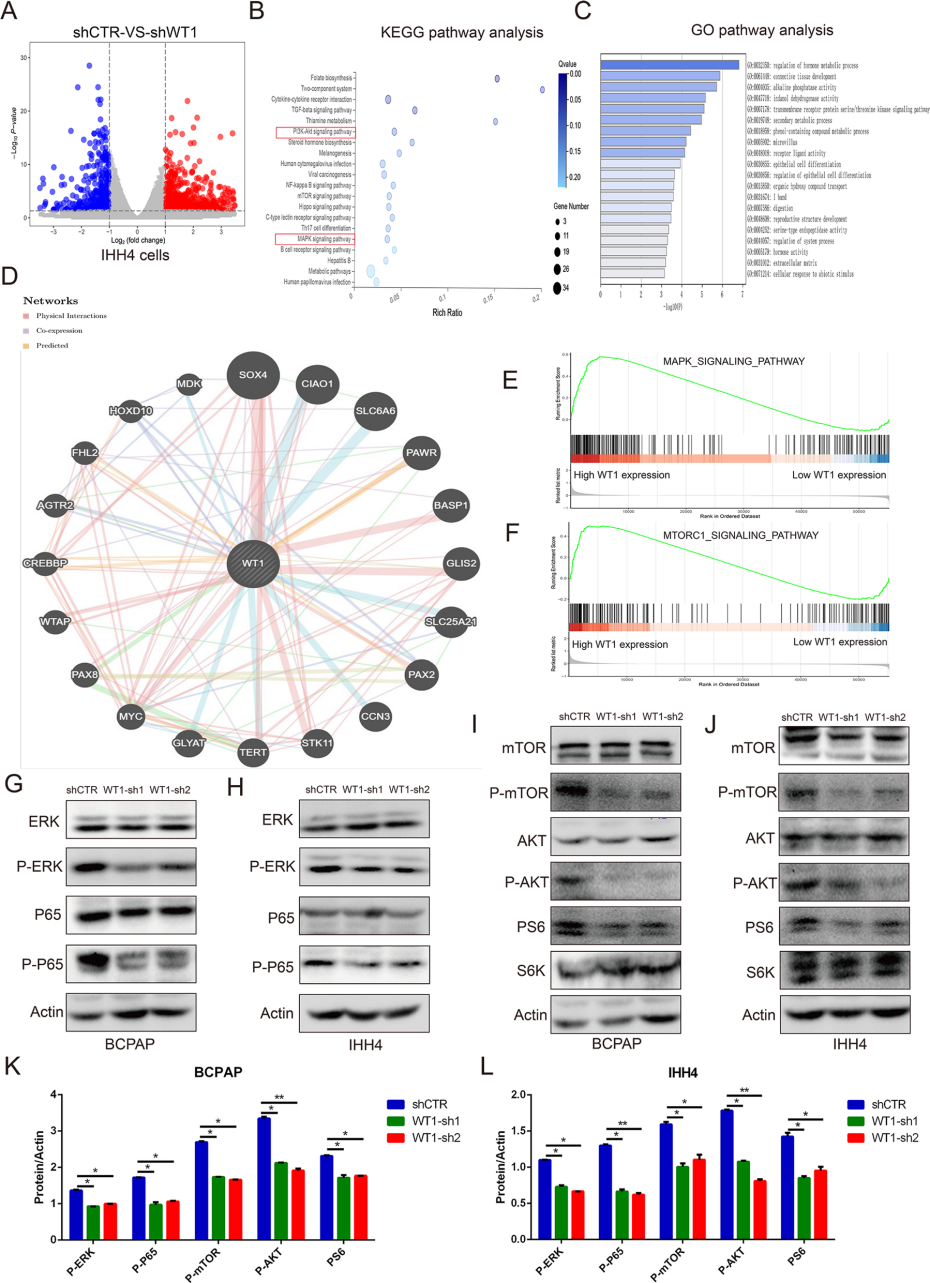
WT1 activated the AKT/mTOR signaling pathway and ERK/P65 signaling pathway in BRAF^V600E^ PTC cells. **A** The differentially expressed genes (DEGs) in IHH4 cells treated with vector lentivirus and shWT1 lentivirus. **B** KEGG analysis indicating the signaling pathways associated with DEGs in IHH4 cells. **C** Go functional analysis showed the 20 most regulatory signaling pathways associated with DEGs in IHH4 cells by Metascape database. **D** The protein–protein interaction (PPI) network indicated the directly regulated molecules of WT1, as determined with the GeneMANIA database. **E**, **F** GSEA showing that higher WT1 expression led to upregulated signaling pathways in thyroid cancer patients. **G**–**J** Western blot analysis showing that WT1 silencing decreased the essential components of AKT/mTOR cascades and the ERK/P65 signaling pathway in IHH4 and BCPAP cells. **K**, **L** Quantification of protein expression of the essential components of AKT/mTOR cascades and the ERK/P65 signaling pathway in WT1-silencing IHH4 and BCPAP cells

The molecular mechanism of WT1 in the progression of BRAF^V600E^ PTC cells was investigated, and the results are presented as a volcano plot showing the differentially expressed genes (DEGs) in IHH4 cells with or without WT1 knocked down (Fig. 6A). A Kyoto Encyclopedia of Genes and Genomes (KEGG) analysis indicated that WT1 is associated with the “PI3K/AKT signaling network”, “mTOR signaling cascades”, “MAPK signaling pathway” and “NF-kappa B signaling pathway” in PTC cells (Fig. 6B). Top 20 go functional analysis indicated “epithelial cell differentiation” and “transmembrane receptor protein serine/threonine kinase signaling pathway” is changed in IHH4 cells with WT1 knocked down (Fig. 6C). Then, a protein–protein interaction (PPI) network was generated using the GeneMANIA database, and it showed the potential regulatory proteins of WT1 (Fig. 6D). Interestingly, A GSEA indicated that WT1 is involved in the “MAPK signaling pathway” and “mTOR signaling pathway” in thyroid cancer patients (Fig. 6E, F). A western blot analysis revealed that knockdown of WT1 expression effectively inhibited the protein expression levels of phosphorylated ERK and phosphorylated P65 in IHH4 and BCPAP cells (Fig. 6G, H). In addition, WT1 inhibition also suppressed the protein levels of phosphorylated AKT, phosphorylated mTOR, and phosphorylated S6 in IHH4 and BCPAP cells (Fig. 6I, J). However, the total protein level of these molecules was not changed in the IHH4 cells or BCPAP cells (Fig. 6G–L). All these results suggest that WT1 activated AKT/mTOR cascades and the ERK/P65 signaling axis in BRAF^V600E^ PTC cells.

### Knockdown of WT1 expression inhibited the growth of PTC tumors expressing BRAF^V600E^

**Fig. 7:**
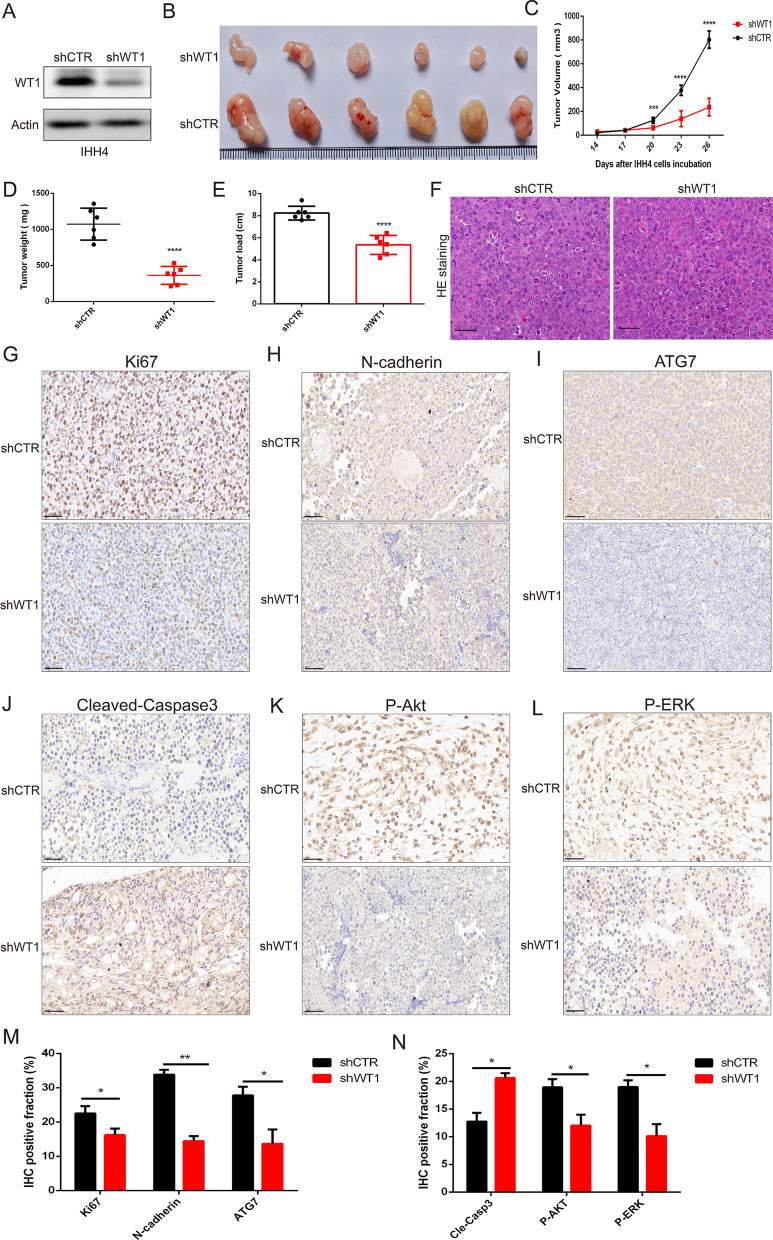
WT1 inhibition attenuates tumor growth of BRAF^V600E^ mutation PTC. **A** Western blot analysis indicated the stable inhibition efficiency of lentiviral WT1 shRNA treatment. **B** The image revealed the tumor volume in the control vector group and WT1 shRNA group. **C** The tumor volume of BRAF^V600E^ PTC xenografts was measured every three days. **D**, **E** The tumor weight and tumor load of BRAF^V600E^ PTC xenografts were determined when tumor-bearing mice were sacrificed. **F** HE staining indicated the pathological features of BRAF^V600E^ PTC xenografts tissue with or wihout WT1 inhibition. **G**–**L** IHC analysis demonstrating that WT1 knockdown regulated the protein expression of Ki67, N-cadherin, ATG7, cleaved caspase 3, P-Akt and P-ERK in BRAF^V600E^ PTC xenograft tissues. **M**, **N** Quantification of immunohistochemistry data of the protein expression of Ki67, N-cadherin, ATG7,cleaved caspase 3, P-Akt and P-ERK in BRAF^V600E^ PTC xenograft tissues. (***P < 0.001, ****P < 0.0001)

To confirm the roles of WT1 in tumor growth of BRAF^V600E^ PTC cells, IHH4 cells were treated with lentiviral WT1 shRNA to stably knockdown WT1 expression, and western blotting was performed to measure the WT1 knockdown efficiency in the IHH4 cells (Fig. 7A). Then, IHH4 cells with or without WT1 were subcutaneously injected into male BALB/c nude mice to establish subcutaneous BRAF^V600E^ PTC xenograft models. Images revealed that knockdown of WT1 expression effectively abolished the growth of BRAF^V600E^ PTC tumors (Fig. 7B). Figure 7C–E shows that silencing WT1 expression notably reduced the tumor volume (Fig. 7C), tumor weight (Fig. 7D) and tumor load (Fig. 7E) in BRAF^V600E^ PTC. HE staining implied that the pathological characteristics of subcutaneous BRAF^V600E^ PTC xenograft tissue with or without WT1 knockdown (Fig. 7F). In addition, IHC assays showed that the protein levels of Ki67, N-cadherin, ATG7, P-Akt and P-ERK were remarkably decreased and that the protein level of cleaved caspase 3 was upregulated upon WT1 inhibition (Fig. 7G–N). Altogether, these results demonstrate that knocking down the WT1 gene significantly suppressed tumor progression and the development of BRAF^V600E^ PTC.

### Validation of the independent predictive ability of WT1 in thyroid cancer patients

**Fig. 8:**
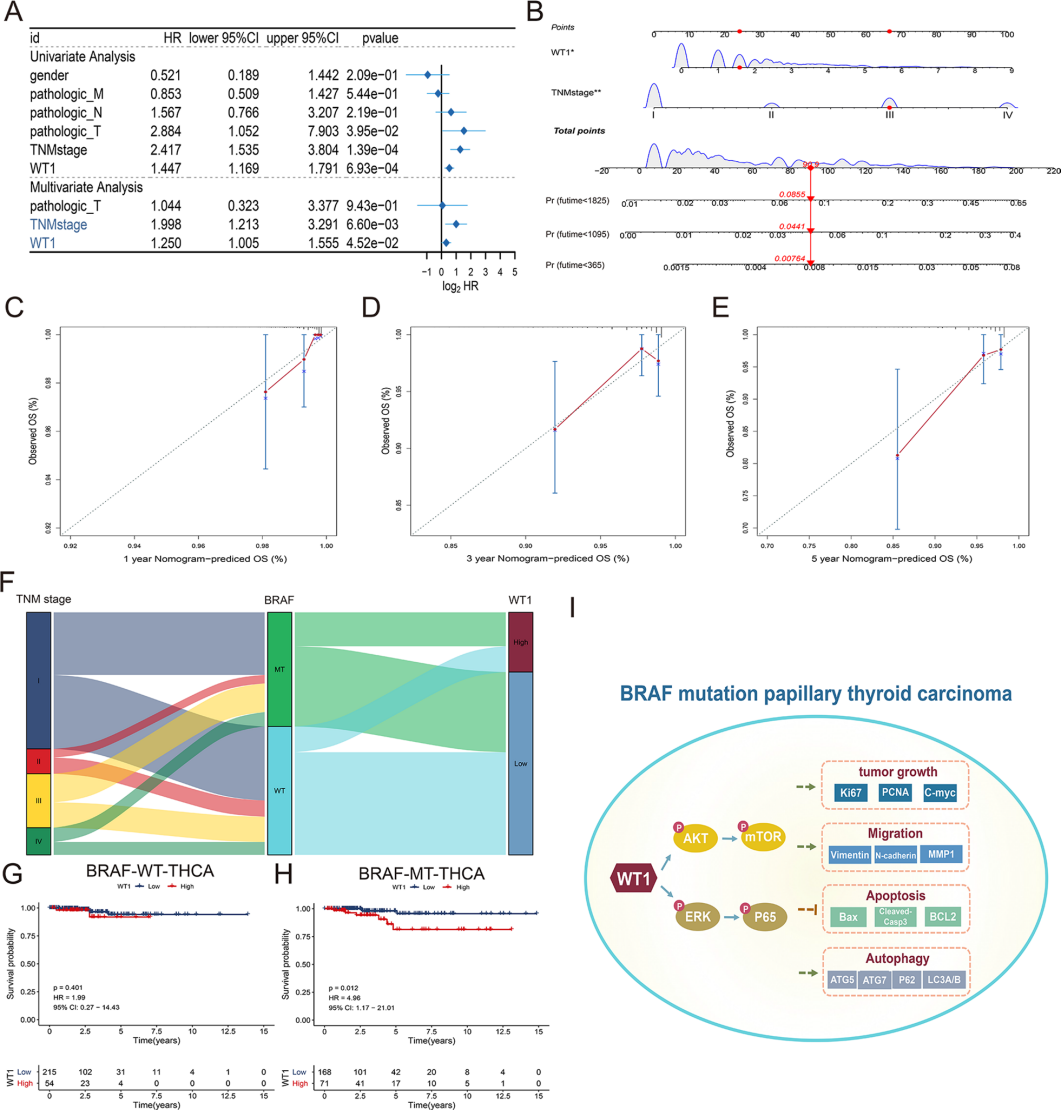
Validation of the independent predictive ability of WT1 in thyroid cancer patients. **A** Forest plot showing the prognostic performance of WT1 and other clinical features in THCA cohort patients in the TCGA database. **B** The nomogram showing the predicted overall survival rate of thyroid cancer patients at 1 year, 3 years and 5 years on the basis of independent prognostic factors including TNM stage and WT1 expression level. **C**–**E** Calibration curves revealing superior consistency of the predictive nomogram model at 1 year, 3 years and 5 years. **F** The relationship between WT1 expression, BRAF mutated status and TNM stage in thyroid cancer patients. **G**, **H** K-M survival analysis implied the prognostic value of WT1 in thyroid cancer patients carrying BRAF mutation or BRAF wild-type. **I** Schematic of mechanism of BRAF-activated WT1 contributes to cancer growth and regulates autophagy and apoptosis in papillary thyroid carcinoma

To determine the predictive value of WT1 in thyroid cancer patients, univariate and multivariate Cox regression analyses were performed to identify the independent prognostic factors through forest plots. The results revealed that TNM stage and WT1 expression levels can be regarded as independent prognostic factors in thyroid cancer patients in the TCGA-THCA cohort (Fig. 8A). Then, TNM stage and WT1 expression levels were used to construct a predictive nomogram of thyroid cancer patients in the TCGA-THCA cohort (Fig. 8B). The calibration curves indicated that the nomogram showed great consistency in predicting overall survival rates at 1, 3 and 5 years (Fig. 8C–E). Sankey plot indicated the relationship between TNM stage, BRAF mutated status and WT1 expression in thyroid cancer patients (Fig. 8F). Next, the prognotic role of WT1 was assessing between BRAF mutated and BRAF wild-type thyroid cancer patients. There was no significant change in survival time compared with high WT1 expression and low WT1 expression in BRAF wild-type thyroid cancer patients (Fig. 8G). However, The KM plot showed higher expression level of WT1 predicted worse overall survival time in thyroid cancer patients carrying BRAF mutation (Fig. 8H). And schematic of mechanism of BRAF-activated WT1 contributes to cancer growth and regulates autophagy and apoptosis in papillary thyroid carcinoma (Fig. 8I). These results suggest that WT1 may function as an independent prognostic factor and is closely associated with the survival time of thyroid cancer patients carrying BRAF^V600E^.

## Discussion

Thyroid papillary carcinoma is a kind of endocrine malignancy, and the morbidity of PTC is gradually increasing. According to previous study, the PTC incidence was 1.80 per 100,000 males and 6.20 per 100,000 in females worldwide [28]. Despite the improvement in current treatments, patients with PTC tend to develop distant metastases or recurrence and have poor prognosis because of complex biological characteristics and unclear pathological mechanisms [29]. In this study, we found that WT1 is highly expressed in PTC patients, and higher WT1 expression is predictive of worse overall survival time. In addition, WT1 also functioned as an independent prognostic factor with high predictive value. This result may help improve the clinical outcomes for PTC patients and indicates possibilities for personalized treatment. BRAF mutations are the most frequent mutation subtypes in PTC patients, and targeting BRAF mutants to develop targeted therapy drugs may be an effective method to overcome PTC [30]. In this study, we comprehensively analyzed the differentially expressed genes in BRAF MT and BRAF WT PTC tissue samples obtained from a TCGA patient cohort. Then, we identified the signaling pathway in PTC patient cells with BRAF mutations. The results indicated that BRAF mutation is closely related to the “epithelial cell proliferation”, “apoptosis” and “selective autophagy” signaling pathways in PTC patients, as determined through a GSEA.

WT1 encodes a zinc-finger transcription factor that also plays oncogenic or tumor suppressor roles at the transcriptional and post-transcriptional levels in various malignancies [15]. The expression level of WT1 is remarkably increased in primary thyroid cancer, and WT1 is regarded as a critical prognostic biomarker related to recurrence-free survival in thyroid cancer patients [31, 32]. However, the regulatory relationship between activated BRAF and WT1 remains unclear. The role of WT1 in BRAF mutated PTC progression and development also needs to be further explored. In this study, we found that WT1 was activated downstream of BRAF in PTC patients. More importantly, knockdown of WT1 expression notably reduced the proliferation and migration of BRAF^V600E^ PTC cells. Silencing WT1 also remarkably decreased the expression levels of PCNA, C-myc and N-cadherin in BRAF^V600E^ PTC cells. In addition, WT1 inhibition significantly suppressed tumor growth of BRAF^V600E^ PTC cells in vivo. These results indicate the important role of WT1 in cancer progression and the development of PTC, in particular, and inhibiting WT1 may serve as a promising treatment strategy against PTC with BRAF^V600E^.

Autophagy is an evolutionarily conserved pathway that ultimately leads to intracellular protein and organelle degradation in lysosomes and is triggered by stress and nutrient deprivation [33]. Apoptosis, also called programmed cell death, is a highly ordered and orchestrated cell death process [34]. Autophagy and apoptosis are two different kinds of programmed cell death and play critical roles in tumorigenesis and cancer progression [35]. In a previous study, triggering apoptosis inhibited cancer cell survival and proliferation [36]. In this study, we found that BRAF mutation is closely associated with “autophagy” and “apoptosis” signaling pathways. WT1 was confirmed as a downstream molecule of BRAF and silencing WT1 expression remarkably promoted apoptosis and inhibited autophagy in PTC cells. These results indicated the oncogenic role of WT1 in regulating the cell death process in BRAF^V600E^ PTC cells. Akt, also known as the PKB (protein kinase B) and mTOR (mammalian target of rapamycin) signaling pathway, is the most frequently activated signaling network in human cancers [37, 38]. Extracellular signal-regulated kinase (ERK) and nuclear factor kappa B (NF-κB) also play essential roles in many biological processes, such as cell growth, tumorigenesis and autophagy and apoptosis [39–42]. More importantly, in a previous study, the BRAF^V600E^ mutant notably activated the MAPK signaling pathway in BRAF-mutant thyroid cancers [43, 44]. In this study, RNA sequence revealed that WT1 is closely associated with PI3K/AKT signaling network, mTOR signaling cascades, MAPK signaling pathway and NF-kappa B signaling pathway. Experiments implied that silencing WT1 expression effectively decreases AKT phosphorylation, mTOR phosphorylation and the downstream protein levels of the mTOR signaling pathway (including S6 phosphorylation) in BRAF^V600E^ PTC cells. Interestingly, WT1 inhibition significantly decreased the protein level of phosphorylated ERK and phosphorylated P65 in BRAF^V600E^ PTC cells. In summary, WT1 contributes to cell proliferation, migration and growth of papillary thyroid carcinoma through augmented activation of the AKT/mTOR axis and ERK/p65 signaling pathway.

In summary, we identified differentially expressed genes and signaling pathways in thyroid cancer patients carrying mutant BRAF. Then, we confirmed that higher WT1 levels were closely related to worse prognosis in thyroid cancer patients with BRAF mutation. In addition, knockdown of WT1 expression remarkably inhibited the proliferation and migration of BRAF^V600E^ PTC cells. WT1 inhibition significantly triggered cell apoptosis and inhibited autophagy in BRAF^V600E^ PTC cells. More importantly, silencing WT1 expression effectively inhibited AKT/mTOR and ERK/P65 signaling in BRAF^V600E^ PTC cells. All these results highlight the biological functions of WT1 and suggest a novel therapeutic strategy for PTC with BRAF^V600E^.

However, there are also some limitations in the current study. First, the RNA sequencing, mutation data and clinical information were obtained primarily from the TCGA database, and more clinical samples are needed to validate the prognostic role of WT1 in BRAF mutated PTC patients. Second, more experiments are needed to confirm the biological functions of WT1 and BRAF^V600E^ in PTC, such as experiments with mice with WT1 conditionally knocked out. Third, the WT1 expression in the BRAF mutated background reduced survival but not in the non-mutated BRAF background, which has aroused our great interest and therefore required further experimental research. Need to be explored in further studies.

## Supplementary Information

**Additional file 1: Figure S1.** The landscape of mutations in thyroid cancer patients as identified through the TCGA database. **(A)** The 15 most frequently mutated genes in thyroid cancer patients in the TCGA database. **(B)** The variant classification, variant type, snv class, variant per case and 10 most frequently mutated genes in thyroid cancer patients.

## Data Availability

The data and material used to support the findings of this study are available from the corresponding author upon request.

## Abbreviations

THCA: Thyroid carcinoma
PTC: Papillary thyroid carcinoma
WT1: Wilms’tumor 1
DEGs: Differentially expressed genes
TCGA: The Cancer Genome Atlas
KEGG: Kyoto Encyclopedia of Genes and Genomes
GO: Gene ontology
FBS: Fetal bovine serum
IHC: Immunohistochemistry
ROC: Receiver operating characteristic curve
PKB: Protein kinase B
mTOR: Mammalian target of rapamycin
PKB: Protein kinase B
ERK: Extracellular signal-regulated kinase
NF-κB: Nuclear factor kappa B
